# Supplementary material for: Genes, Structural, and Biochemical Characterization of Four Chlorophyllases from Solanum lycopersicum
Source: Int J Mol Sci. 2022 Oct 3;23(19):11716. doi: 10.3390/ijms231911716 (PMC9570282; doi:10.3390/ijms231911716)
Supplement: Supplementary file 1 [file ijms-23-11716-s001.zip › ijms-1936521-supplementary.pdf]

Table S1. List of primers used in this study.

**Experiment**	**Primers name**	**Oligonucleotides sequences**
	SlCLH1-F	5-ggatttcagaattcggatccATGGTGATAACTAGTACTTCCTC-3
	SlCLH1 ΔN-F	5-ggatttcagaattcggatccATGACTGTTTTTGATATTGGAAATTAC-3
	SlCLH1-R	5-atcgaattcctgcaggtcgacGACAAGAAAATCAATATCTTGGAGC-3
	SlCLH2-F	5-ggatttcagaattcggatccATGCATGGCAATTGGCAAAG-3
Protein Expression	SlCLH2 ΔN-F	5-ggatttcagaattcggatcc ATGTCATCAATTTTCGAAGTAGGT-3
in *E. coli*	SlCLH2-R	5-atcgaattcctgcaggtcgacATCCTTGATAGATATAACTGGATCA-3
	SlCLH3-F	5-ggatttcagaattcggatccATGGTGATAACTTACTCTTCTTC-3
	SlCLH3 ΔN-F	5-ggatttcagaattcggatcc ATGACAATTTTTGACATTGGAAATTAT-3
	SlCLH3-R	5-atcgaattcctgcaggtcgacCATTCGAAAATCCACATCTCG-3
	SlCLH4-F	5-ggatttcagaattcggatccATGGAGAAGGTAAGGGATGATGG-3

	SlCLH4-R1	5-CCACAGATGCATCTTTGGCTACTCGAATGTGAAATAGTGTACTCTTCGATAG-3
	SlCLH4-R2	5-CTTATTTCTTTTTTCAAATCCGCCACAGATGCATCTTTGGCTAC-3
	SlCLH4-R3	5-atcgaattcctgcag<u>gtcgac</u>CTTATTTCTTTTTTCAAATCCGCCAC-3
Site-Directed Mutagenesis	SlCLH1(S135A)-F	5-AACTTTTCCCCCACG**TGC**ATGGCCTGCTAGTCCAAGTTTC-3
	SlCLH1(S135A)-R	5-AT**GCA**CGTGGGGGAAAAGTTGCATTTTCGCTAGCTCTTGG-3
	SlCLH1(D162N)-F	5-CATCCACAGG**GTT**AACACCGATCAACGCTGAAAATTTCAG-3
	SlCLH1(D162N)-R	5-CGGTGTT**AAC**CCTGTGGATGGAATGGAGAAAGGAAAGCAA-3
	SlCLH1(H239A)-F	5-CATATCATT**TGC**TCCATAATCCTTTGCAACAAAGTAACAAGC-3
	SlCLH1(H239A)-R	5-GGATTATGG**AGC**AAATGATATGTTAGATGAGGAGACCAAAGG-3
Real-time PCR	SlCLH1-F	5-CATGGCTTCATTGTTGTTGCTCC-3
	SlCLH1-R	5-GCCTGCTAGTCCAAGTTTCGTC-3
	SlCLH2-F	5-GGCTTCATGCTCCAACCTAG-3
	SlCLH2-R	5-CGGCAGTACGGATTTGAGG-3

	SlCLH3-F	5-CTTCTCCTCCAAAGCCACTC-3
	SlCLH3-R	5-GTAATCCGTCAGGTAACCAGTTT-3
	SlCLH4-F	5-ATTCCGCCTCCAACCCGACT-3
	SlCLH4-R	5-GCAGCACAGAGTTGAGCTTCTTAGC-3
	SlEF-1α-F	5-AGATGGTCAGACCCGTGAAC-3
	SlEF-1α-R	5-TGGAGTACTTGGGGGTGGTA-3

Note: Site-directed mutagenesis introduced are shown in red and bold. *BamH* I and *Sal* I restriction sites are underlined.

Table S2. Pairwise Identity of the Amino Acid Sequence between SlCLHs and Other Plant Chlases.

	CrCLH	SlCLH2	SlCLH4	ClCLH	AtCLH1	GbCLH	SlCLH1	SlCLH3
CrCLH	100	18.75	14.48	19.76	20.12	16.62	17.26	16.57
SlCLH2		100	53.11	33.83	37.85	33.24	38.61	39.63
SlCLH4			100	30.54	34.17	31.44	31.74	32.12
ClCLH				100	39.88	36.39	37.06	39.83
AtCLH1					100	40.00	40.37	39.33
GbCLH						100	47.23	49.13
SlCLH1							100	73.90
SlCLH3								100

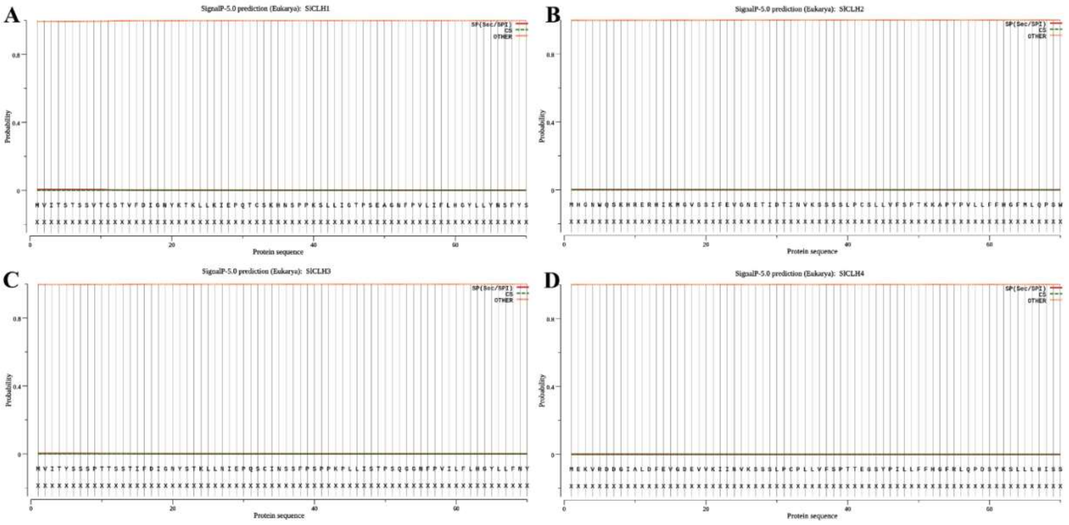

Figure S1. Analysis   the predicted N-terminal signal peptide sequence of SlCLH1 (A), SlCLH2 (B), SlCLH3 (C) and SlCLH4 (D) by using SignalP-5.0 server. Based on the D-cutoff value, no signal peptide was found in four SlCLHs. C score, S score, and Y score are depicted as pink, green, and blue respectively.

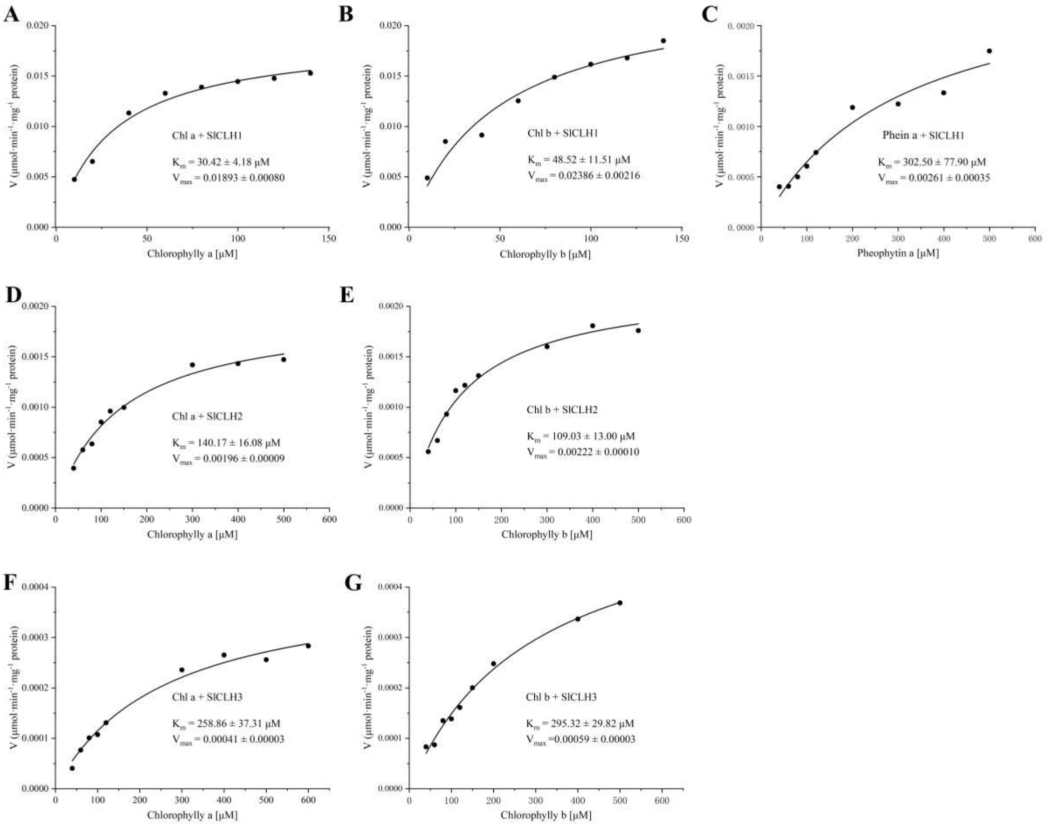

Figure S2. Non-linear regression plots for kinetic parameter assays of SlCLHs. Activity of MBP-SlCLH1 on chlorophyll a (A), chlorophyll b (B) and pheophytin a (C) followed Michaelis-Menten kinetics. Activity of MBP-SlCLH2 on chlorophyll a (D) and chlorophyll b (E), MBP-SlCLH3 on chlorophyll a (F) and chlorophyll b (G), followed Michaelis-Menten kinetics.
